# Targeting Refractory Triple-Negative Breast Cancer with Sacituzumab Govitecan: A New Era in Precision Medicine

**DOI:** 10.3390/cells13242126

**Published:** 2024-12-22

**Authors:** Saif Khan, Suresh Babu Jandrajupalli, Nashwa Zaki Ali Bushara, Rama Devi Patel Raja, Shadab Mirza, Kuldeep Sharma, Rajan Verma, Ashish Kumar, Mohtashim Lohani

**Affiliations:** 1 Department of Basic Dental and Medical Sciences, College of Dentistry, University of Ha’il, Ha’il 55473, Saudi Arabia; sf.khan@uoh.edu.sa (S.K.); s.mirza@uoh.edu.sa (S.M.); 2 Department of Preventive Dental Sciences, College of Dentistry, University of Ha’il, Ha’il 55473, Saudi Arabia; s.jandrajupalli@uoh.edu.sa (S.B.J.); n.bushara@uoh.edu.sa (N.Z.A.B.); 3 Department of Biology, College of Science, University of Ha’il, Ha’il 55473, Saudi Arabia; r.patel@uoh.edu.sa; 4 Centre of Research Impact and Outcome, Chitkara University, Rajpura 140401, India; kuldeep.sharma@chitkara.edu.in; 5 Chitkara Center for Research and Development, Chitkara University, Baddi 174103, India; rajan1708verma@gmail.com; 6 Department of Mechanical Engineering, Institute of Aeronautical Engineering, Hyderabad 500043, India; ashishrahul79@gmail.com; 7 Division of Research and Development, Lovely Professional University, Phagwara 144411, India; 8 Department of Nursing, College of Nursing and Health Sciences, Jazan University, Jazan 45142, Saudi Arabia

**Keywords:** triple-negative breast cancer (TNBC), antibody–drug conjugates, sacituzumab govitecan, neutropenia, ADC sequencing, resistance

## Abstract

Advanced triple-negative breast cancer (TNBC) has poorer outcomes due to its aggressive behavior and restricted therapeutic options. While therapies like checkpoint inhibitors and PARP inhibitors offer some benefits, chemotherapy remains ineffective beyond the first line of treatment. Antibody–drug conjugates (ADCs) like sacituzumab govitecan-hziy (SG) represent a significant advancement. SG combines SN-38, an irinotecan derivative, with a Trop-2-targeting antibody via a pH-sensitive linking moiety, achieving a good drug:antibody ratio. In a phase I-II study involving metastatic TNBC (mTNBC) individuals, SG achieved an overall response rate of 33.3% and a median response period of 7.7 months. The phase III ASCENT trial demonstrated SG’s efficacy in relapsed or refractory TNBC, improving median progression-free survival and median overall survival compared to chemotherapy. Common side effects include neutropenia, nausea, and fatigue. This article highlights the clinical potential, pharmacokinetics, safety profile, and resistance mechanisms of SG along with key ongoing clinical trials, emphasizing its role in managing refractory mTNBC, especially in third-line therapy. The review also discusses current strategies for managing adverse reactions and sequencing ADC treatments in clinical practice, along with the predicted basis of resistance. The optimal sequencing of SG relative to other ADCs, such as trastuzumab deruxtecan or T-DXd, remains an evolving question, especially as newer agents with distinct mechanisms of action and safety profiles enter the field. Further research is essential to establish evidence-based strategies for sequencing SG and addressing disease progression post-ADC therapy.

## 1. Triple-Negative Breast Cancer 

Breast cancer (BC) stands as the most prevalent neoplasm affecting females globally, with over 2.3 million freshly diagnosed cases documented in 2022 [1,2]. Due to its heterogeneous nature, it remains the second largest contributor to cancer-associated fatalities [3]. Representing ~15–20% of total invasive BCs, Triple-Negative Breast Cancer (TNBC) differs significantly from other forms due to its distinct molecular and clinical hallmarks [4,5]. TNBC lacks estrogen, progesterone, and HER2 (human epidermal growth factor 2) receptors [6]. This receptor-negative status excludes TNBC patients from benefiting from hormonal or HER2-targeted therapeutic agents. As a result, TNBC is managed with chemotherapy before surgery, often referred to as (neo)-adjuvant chemotherapy (NAC) [7,8], which remains the standard care, especially in early-stage disease. The overall sensitivity of TNBC to NAC is strongly related to long-lasting clinical efficacy, as NAC lowers the tumor load and nodal involvement [9]. Furthermore, median survival for advanced or metastatic TNBC (mTNBC) is around 12–14 months, with significant symptoms impacting quality of life [10]. Current single-agent chemotherapies for recurrent locally advanced TNBC and mTNBC show low response rates (5–10%) and progression-free survival (PFS) rates of mere 2–3 months [11].

### 1.1. Clinical Characteristics of TNBC

TNBC, often diagnosed in younger individuals, is highly aggressive, with a greater risk of early recurrence, poor outcomes, and frequent metastasis to the lungs, lymph nodes, and brain, leading to a survival rate of fewer than two years [12,13,14]. Secondary relapse is three times more likely in TNBC patients within five years of diagnosis but drops to less than 3% thereafter [15]. TNBC tumors are typically Grade 3, exhibit rapid growth with high Ki-67 levels [16], and are strongly linked to *BRCA1/BRCA2* mutations, found in 10.6–30.9% of cases [17,18]. International variations in mutation prevalence highlight the need for population-specific risk assessment tools, as existing models often underestimate risk in non-Caucasian populations.

### 1.2. Molecular Subtypes and Heterogeneity of TNBC

Molecular heterogeneity of been progressively explored through advanced transcriptomics. Lehmann and coworkers initially conducted a comprehensive analysis of 21 publicly available microarray datasets, identifying seven distinct TNBC subtypes: basal-like 1 (BL1), basal-like 2 (BL2), immunomodulatory (IM), mesenchymal (M), mesenchymal-stem-like (MSL), luminal androgen receptor (LAR), and an unstable subtype (UNS) [19]. These classifications relied on the manifestation of key markers like ESR1, PGR, and ERBB2 and are defined by unique molecular signatures, including patterns of gene expression, mutations, and polymorphism in copy numbers [7,20].

**Figure 1:**
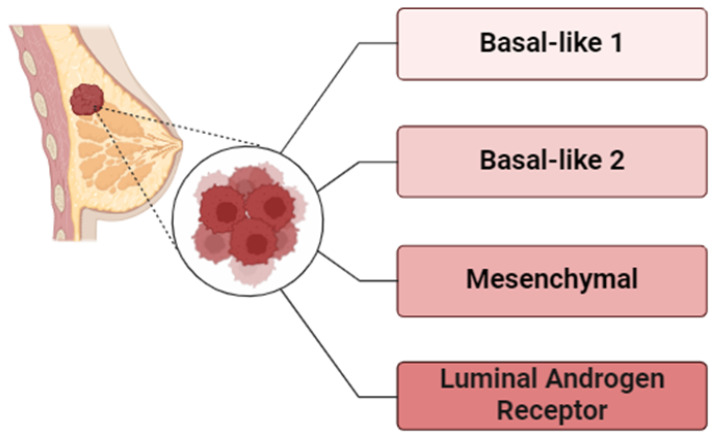
Molecular classification of Triple-negative breast cancer (TNBC) as reported by [21].

In 2016, Lehmann and colleagues revisited the classification system (Figure 1), finding that some of the earlier subtypes, particularly IM and MSL, were prejudiced by the prevalence of cancer-associated stromal cells and tumor-infiltrating lymphocytes (TILs), which complicated their initial definitions. This led to a refined four-subtype classification—BL1, BL2, M, and LAR—commonly known as TNBCtype4 [21]. Each of these subtypes exhibits distinct clinical outcomes, including variations in response to chemotherapy and patterns of recurrence. For instance, BL1 tends to have the highest response rates (65.6%) to NAC, while LAR tumors often involve local lymph nodes and are highly prone to bone metastases [22]. The recently developed IO Score is a 27-gene immuno-oncology (IO) descriptor that was developed to parse out immune-responsive TNBCs across all the TNBC types described by Lehmann. This IO score is linked with improved responses to immunotherapy in TNBC [23].

Single-cell RNA sequencing studies have further complicated this picture, showing that most TNBC tumors contain multiple subtypes within a single mass, revealing the intratumoral heterogeneity that bulk sequencing methods fail to capture. This finding suggests that more nuanced and granular approaches, like single-cell analyses, are required to fully understand the biological processes at play and improve treatment strategies [24]. Further, variations in the intra- and inter-tumoral diversity across TNBC and ER^+^ BC may partially clarify the pitfalls in applying commercial gene-expression tests for prognosis in TNBC.

### 1.3. Treatment Challenges

As TNBC cells lack hormone receptors, standard endocrine therapies and targeted treatments are ineffective [25]. Drug chemotherapy is typically the stronghold of managing TNBC, particularly in early-stage disease, where it is often administered as (neo)-adjuvant chemotherapy (NAC), given before surgery to shrink tumors and reduce nodal involvement. NAC is the most widely accepted course of therapy for stage II–III TNBC [26,27,28]. Traditionally, NAC involves treatment with anthracyclines, cyclophosphamides, and paclitaxel, resulting in a pathologic complete response (pCR) rate of 35–45% [29,30,31]. Additionally, clinical trial designs like I-SPY 2 (NCT01042379) [32] and ARTEMIS (ISRCTN15384496) [33] aim to expedite the development of effective targeted treatments for NAC. However, despite its efficacy in reducing tumor burden, ~50% of TNBC patients exhibit residual disease after NAC, indicating the persistence of cancer cells.

**Figure 2:**
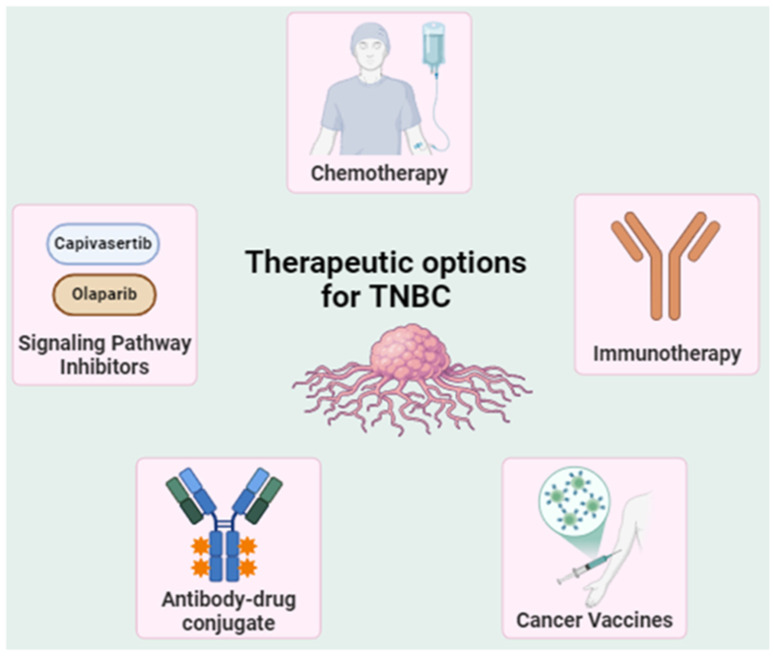
Treatment options for triple-negative breast cancer (TNBC).

Over the past decade, advancements such as PARP inhibitors (PARPi), AKT inhibitors, and immune checkpoint inhibitors (ICIs) have provided hope by targeting specific molecular pathways or enhancing immune responses (Figure 2). For example, PARPi like olaparib (Lynparza) and talazoparib (Talzenna) offer significant benefits in BRCA1/2-mutated TNBC [34], while AKT inhibitors (e.g., capivasertib and ipatasertib) show promise in targeting dysregulated PI3K/AKT pathways [35,36,37]. Olaparib is now approved as an adjunctive chemotherapy for those with germline BRCA-mutated (gBRCAm) HER2 BC pre-treated with NAC. ICIs, particularly PD-1/PD-L1 inhibitors like pembrolizumab, have demonstrated efficacy in improving PFS and overall survival (OS) in PD-L1^+^ TNBC if coupled with chemotherapeutic agents [38]. In clinical trials like KEYNOTE-355 and KEYNOTE-522, the pCR was considerably greater (64.8% vs. 51.2%) with longer OS (23.0 vs. 16.1 months) than NAC alone [39,40].

Therapeutic approaches such as adoptive cell transfer (ACT) and chimeric antigen receptor (CAR) T-cell therapy are being explored for their promising clinical efficacy and improved cancer eradication [41]. Furthermore, autogene cevumeran (RO7198457), an RNA-lipoplex targeting neoantigens, combined with atezolizumab, demonstrated a manageable safety profile and induced vigorous neoantigen-specific T-cell responses in a phase Ib trial (NCT03289962), achieving an 8% objective response rate (ORR) and 49% stable disease [42].

Precision medicine in TNBC tailors treatments to the tumor’s molecular profile, aiming for better outcomes and reduced side effects. Recent advancements in single-cell transcriptomics have uncovered TNBC’s complexity, revealing distinct tumor and immune cell populations with unique pathways and therapeutic susceptibilities. This enables the identification of actionable targets and insights into tumor subclonal dynamics, aiding in personalized treatment strategies. Further, genomic profiling, like NGS, detects mutations, guiding tailored therapies with enhanced efficacy and diminished adverse effects (AEs) [26].

In this context, antibody–drug conjugates (ADCs) present a transformative stratagem to TNBC management. ADCs combine the precision of monoclonal antibodies (MAb) targeting specific tumor-associated antigens with the cytotoxic power of chemotherapy, allowing for direct release of therapeutics to neoplasms while minimizing damage to normal tissues. This “Trojan horse” mechanism is particularly valuable in cancers like TNBC, where heterogeneity and resistance to therapy are common. Among the emerging ADCs, sacituzumab govitecan (SG) has garnered significant attention and regulatory approval for its role in addressing the unmet needs of patients with metastatic TNBC (mTNBC). SG targets trophoblast cell surface antigen 2 (Trop-2), a protein highly expressed in TNBC cells, and releases a highly active topoisomerase-I (TOPO-I) inhibitor, directly to tumor sites. This targeted delivery minimizes systemic toxicity while enhancing therapeutic efficacy. 

SG, endorsed by the FDA on 22 April 2020 for mTNBC [43], has shown significant clinical benefits, particularly for heavily pretreated patients who have exhausted other options. SG combines targeted delivery of chemotherapy with manageable side effects. Phase II/III trials demonstrate notable improvements in PFS and OS, although further studies are needed to confirm its efficacy and explore Trop-2′s role in malignancies. SG exemplifies the potential of ADCs to revolutionize TNBC care, offering a more selective, less toxic treatment option and paving the way for advancements across oncology. As ADC technology continues to advance, therapies like SG may redefine the standard of care for this challenging malignancy, bridging critical gaps in therapeutic care and improving the quality of life for patients worldwide. This review examines the development, mechanism of action, pharmacokinetics, AEs, safety profile, and clinical efficacy of SG, while emphasizing the need for further trials and clinical practice.

## 2. Sacituzumab Govitecan, a Trop-2–Directed Antibody–Drug Conjugate

In order to develop a targeted therapeutic intervention, a humanized monoclonal immunoglobulin (hRS7, Sacituzumab, [44]) was linked with a potent chemotherapeutic molecule, SN-38 (govitecan), a derivative of irinotecan (CPT-11), TOPO-I inhibitor, with the help of a proprietary hydrolyzable linking agent to form Sacituzumab govitecan (SG, IMMU-132, Trodelvy^TM^) (Gilead Sciences, Foster City, CA, USA), a first-in-class third-generation ADC [45,46]. Because of its unique design, the medication can be precisely delivered to breast cancer cells without endangering healthy tissues. Table 1 summarizes the essential features of SG.

### 2.1. The Discovery and Development of Sacituzumab Govitecan

#### 2.1.1. Trop-2 and Its Expression Pattern

Primarily detected in trophoblasts [47], Trop-2, a trans-membrane glycoprotein receptor expressed by *TACSTD2* gene loci on chr 1p32, has been classified under various names including tumor-associated calcium signal transducer 2 (TACSTD2), membrane component chromosome 1 surface marker 1 (M1S1), gastrointestinal antigen 733-1 (GA733-1), and epithelial glycoprotein-1 (EGP-1) [48,49]. 

Trop-2 plays an essential part in tumorigenesis, especially in TNBC, by promoting epithelial-mesenchymal transition (EMT), distant metastasis, and resistance to programmed cell death. Trop-2 is related to numerous aggressive malignant characteristics, including lymph node inflammation, metastasis, and poor OS [50]. Its overexpression is observed in 95% of TNBC cases and 88% of metastatic TNBC [51,52], making it a significant therapeutic target [53,54]. Furthermore, circulating neoplastic cells that constitutively display Trop-2 on their exterior, serve as excellent biomarkers for EMT as well as distant metastasis [55].

ADC-based targeted therapies have emerged for Trop-2-expressing TNBC, although their effectiveness is limited, with response rates not exceeding 35% [53,56,57]. The ASCENT trial demonstrated improved PFS and OS in individuals over-expressing Trop-2, emphasizing the importance of this receptor as a therapeutic marker [58,59,60,61]. Trop-2′s role in TNBC therapy is complex, with ADC trials largely targeting patients lacking other treatment options, rather than those with high Trop-2 expression [62]. Further, its expression in normal tissues raises concerns about side effects [47,63], since just 54.4% of TNBC patients express Trop-2 [64]. Fluctuations in Trop-2 expression induced by EMT complicate its role as a biomarker.

#### 2.1.2. RS7 Monoclonal Antibody

The RS7 murine monoclonal antibody, initially generated by Stein et al. [65] to target human non-small-cell lung cancer (NSCLC), also binds to a wide array of neoplasms, notably BC cells, with some binding observed in non-cancerous cells [66,67]. It targets a 46 kDa glycoprotein receptor, phosphorylated at serine-303 by protein kinase C, which is reduced to 35 kDa upon deglycosylation. Later identified as epithelial glycoprotein-1 (EGP-1) [66,68], it is an essential component of the intracellular ERK/MAPK signaling cascade [69]. RS7, known for its rapid internalization by cells [65], shows promise as a vehicle for radiometabolic therapy. Studies using radioiodine-labeled RS7 in a prostate tumor model via SPECT support its potential for targeting cancer cells in both imaging and treatment [70].

#### 2.1.3. SN-38, a Camptothecin Derivative

SN-38 (7-ethyl-10-hydroxycamptothecin), a semi-synthetic analogue of camptothecin, and the bioactive metabolite of irinotecan, was selected for its well-established clinical efficacy. Camptothecins target cancer cells by engaging with TOPO-I, resulting in the formation of double-stranded DNA lesions in the S-phase of cell cycle [71,72]. Irinotecan, a precursor drug of SN-38, is highly active against solid cancers, particularly mTNBC [73]. The cytochrome P450 3A4 (CYP3A4) enzyme converts it into two inert compounds, 7-ethyl-10-[4-N-(5-amino-pentanoic acid)-1-piperidino]carbonyl oxycamptothecin (APC) and 7-ethyl-10-(4-amino-1-piperidino)carbonyl oxycamptothecin (NPC) [74].

SN-38 has an IC_50_ of 1.0–6.0 nM, but its hydrophobicity and limited coupling sites make it challenging to formulate and contribute to poor tolerability [75]. Modifications in the structure, like glucuronidation and lactone ring opening, reduce its potency. To improve solubility, a dipiperidino side chain was added at C_10_, creating irinotecan. Once this carbamate side chain cleaved in the liver carboxylesterases, SN-38, up to 1000 times more potent than irinotecan, is released but quickly becomes glucuronidated by uridine diphosphate-glucuronosyl transferase 1A1 (UGT1A1), affecting its efficacy [76]. SN-38 and its glucuronide (SN-38G) are subsequently lost into the bile and intestines. Since UGT1A1 is associated with the metabolism of SN-38, medications that influence UGT1A1 enzyme function might boost or shorten the intake of SN-38 [77].

#### 2.1.4. Conjugation of RS7 with SN-38

**Figure 3:**
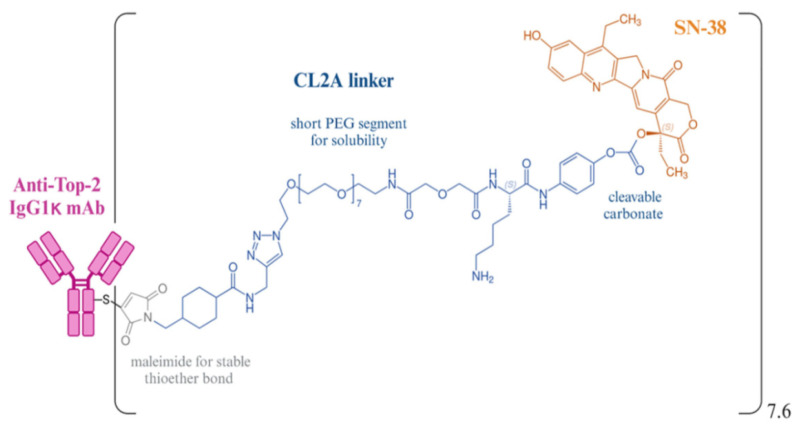
Structure of SG. A scheme highlighting the chemical arrangement of the link between SN-38 (orange) and the hRS7 antibody (pink) with the CL2A linker (blue). Republished with permission from [62], doi: 10.3389/fimmu.2024.1447280; under Creative Commons Attribution License, CC-BY, https://creativecommons.org/licenses/by/4.0/. © 2024 Rossi, Turati, Rosato, and Carpanese.

Researchers optimized SN-38′s performance by attaching linkers to the C10 and C20 positions, using polyethylene glycol (PEG) to enhance solubility and reduce aggregation [45,46,78]. A maleimide moiety was introduced to link SN-38 with the sulfhydryl side chain on the IgG, creating a durable thioether linkage. Some linkers included a cathepsin B cleavage site (Phe-Lys) for tumor-specific release and a pH-sensitive benzyl carbonate bond to release the drug in acidic environments [45]. The original linker (CL2) exhibited intermediate serum stability (1–2 days) [78], and was later modified to a CL2A derivative by removing phenylalanine at the cathepsin B cleavage site, improving product quality and yield without affecting SN-38 release [46,79]. The CL2A linking agent, which contains a short PEG chain enhances solubility and couples SN-38 at the 20^th^ location of the lactone ring, stabilizing it from converting to its less potent carboxylate derivative (Figure 3). Further, the 10th residue of SN-38 becomes shielded against glucuronidation after its attachment to RS7, allowing SN-38 to remain in its most active state until release.

The conjugation process involved linking the SN-38 complex to mildly reduced IgG, targeting eight specific thiol sites (two per heavy chain in the hinge region and four in the C_H_^1^-C_L_region) without disrupting the antibody’s antigen-binding ability [46]. Liquid chromatography-mass spectroscopy confirmed site-specific coupling away from the antigen-binding regions. Pharmacokinetic studies indicated the conjugate cleared similarly to native IgG, with stable chain association despite disulfide bond disruption. The conjugate maintained its immunoreactivity, and in mouse studies, the IgG component cleared from the body at a rate similar to that of the native IgG. SN-38 was released as expected, with none found after 72 h, suggesting a minimal risk of off-target toxicity [75].

### 2.2. Mode of Action of Sacituzumab Govitecan

**Figure 4:**
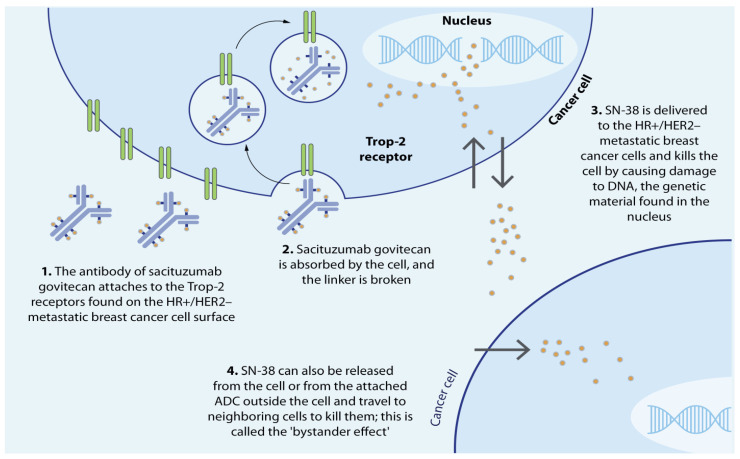
Mechanism of action of Sacituzumab govitecan (Reproduced from [60], Future Oncology, doi: 10.2217/fon-2021-0868; under the Attribution-NoDerivatives 4.0 Unported License, https://creativecommons.org/licenses/by-nd/4.0/). © 2021 The Authors.

After administration, the hRS7 antibody binds to the Trop-2 receptor, facilitating internalization of the conjugate via endocytosis [80]. The acidic endosomal environment triggers hydrolysis of the pH-sensitive linking agent, releasing the active drug into the cytosol when fused with the lysosomal compartment [81]. SN-38 binds and stabilizes TOPO-IB, leading to the formation of a stable SN-38-TOPO-IB-DNA complex, which prevents the repair of DNA lesions [82,83]. As DNA replication proceeds, this results in irreversible single-stranded breaks, leading to cell death [59]. In addition, the membrane-permeable SN-38 is released into the tumor niche, inducing cytotoxic activity in nearby Trop-2^+^ and Trop-2^-^ tumor cells through the bystander effect [84] (Figure 4), thus overcoming the heterogeneity of Trop-2 expression [46,85,86]. The pH-sensitive linker further facilitates SN-38 release in the acidic tumor microenvironment (TME), enabling targeted cell death of adjacent cells. This mechanism contrasts with anthracyclines, which target the topoisomerase II (TOPO-II) enzyme that repairs double-stranded DNA lesions. Nonetheless, both SN-38 and anthracyclines induce cell death through p53-mediated apoptosis, despite targeting different topoisomerases [87].

### 2.3. Pharmacokinetics (PK) Profile of Sacituzumab Govitecan

The phase I/II basket trial and PK studies of SG demonstrated consistent efficacy and tolerability across various settings. Based on phase I findings, a dose of 10 mg/kg was chosen for phase II. PK analyses from IMMU-132-01, TROPHY-U-01, and ASCENT trials showed no drug or SN-38 accumulation, underscoring its favorable PK profile [88]. SG displayed enhanced Trop-2 binding (K_d_: 0.26 ± 0.14 nM) compared to the naked antibody hRS7 (K_d_: 0.51 ± 0.04 nM), delivering higher SN-38 levels with reduced toxicity [83]. Approximately 50% of the drug was released daily [89,90], resulting in prolonged intratumoral SN-38 retention (3 days vs. 8 h for irinotecan) [75].

Population PK (popPK) modeling indicated that SG metabolism was unaffected by age, sex, race, renal/hepatic function, Trop-2 expression, or UGT1A1 polymorphisms [91,92]. Higher SG exposure was linked to improved outcomes, such as longer PFS, OS, and better response rates, though it also increased the risk of adverse effects like diarrhea, neutropenia, and hypersensitivity [93]. SG exhibited a median elimination half-life (t½) of 16 h (Table 2) [94], maintaining significant plasma drug levels during circulation and supporting its superior efficacy compared to irinotecan.

SG achieved a maximum serum concentration (C_max_) of 239,000 ng/mL and an area under the curve (AUC_0–168_) of 5,640,000 ng·h/mL, while SN-38′s C_max_ and AUC_0–168_ were 98 ng/mL and 3696 ng·h/mL, respectively. Less than 5% of SN-38 was free in serum, reflecting strong conjugate binding. SG clearance was unaffected by cancer type, while free SN-38 levels had no correlation with neutropenia severity, and no antibody responses to the conjugate were reported [89].

UGT1A1 metabolizes SN-38, and its glucuronide product (SN-38G) is detected in blood. However, conjugation of SN-38 with RS7 protects it from glucuronidation, leading to a lower plasma SN-38G:SN-38 ratio for SG (1:5) vs. irinotecan (>4:1). This suggests the need for further research to alleviate AEs in subjects exhibiting diminished UGT1A1a function [56,75]. No significant drug–drug interactions have been reported for SG; however, UGT1A1 inducers, inhibitors, or genetic variants may influence systemic SN-38 exposure as well as patient outcomes.

### 2.4. Clinical Efficacy of Sacituzumab Govitecan

The effectiveness of SG was first studied in the phase I/II multicenter basket trial (IMMU-132-01) in 108 mTNBC individuals who had undergone prior treatments, at an intravenous dosage of 10 mg/kg on day 1 and day 8 of each 21-day treatment phase. The median duration of treatment and median time to response were 5.1 and 2.0 months (mo), respectively. The investigation showed a 33% ORR (95% confidence interval [CI], 24.6–43.1), with a median PFS (mPFS) and OS of 5.5 and 13.0 mo, respectively. Durable responses were noted, with a median response duration of 7.7 mo (95% CI, 4.9–10.8). After six and twelve months of therapy, the calculated probability of a response is 27.0% and 59.7%, respectively. Subgroup analysis in ER^+^/HER2^−^ cancer individuals showed a 31.5% response rate, with mPFS and median OS (mOS) of 5.5 mo (95% CI, 4.1–6.3) and 12 mo (95% CI, 11.2–13.7), respectively. The median duration of response (mDOR) was 8.7 mo [56,57,95]. On the basis of the clinical outcomes of the trial, SG (Immunomedics, Inc.) received FDA authorization for use in individuals with mTNBC who had previously undergone no less than two treatments in a metastatic setting [96].

In heavily pretreated mTNBC individuals, SG exhibited a 30% ORR, 8.9-month mDOR, and 46% clinical benefit rate (CBR). The mPFS and mOS were 6.0 and 16.6 mo, respectively. Frequent grade ≥ 3 AEs were neutropenia, leukopenia, anemia, and febrile neutropenia. Trop-2 levels were strong in 88% of tissues, and no neutralizing antibodies were noticed following repeated therapy [95].

The phase III ASCENT trial involving 468 patients verified the effectiveness of SG in a population with relapsed or refractory TNBC, leading to its FDA approval (Table 3). SG significantly improved mPFS to 5.6 mo vs. 1.7 mo with physician’s choice of single-agent treatment (TPC) (95% CI, 4.3–6.3 vs. 1.5–2.6; hazard ratio [HR], 0.41), and mOS was 12.1 vs. 6.7 mo (95% CI, 10.7–14.0 vs. 5.8–7.7; HR, 0.48). Radiological responses (ORR) were reported in 35% of individuals administered with SG in comparison with 5% in the chemotherapy group. However, SG was linked to greater frequencies of Grade 3 or higher adverse reactions, particularly neutropenia [60,61].

A phase II TROPiCS-02 trial (Table 3) demonstrated significant benefits for HR^+^/HER2^−^ mBC individuals treated with the conjugate. At the July 1, 2022, data cutoff, the study involving 543 patients found that SG improved OS (14.4 mo) vs. chemotherapy (11.2 mo) with a higher ORR (21% vs. 14%). Additionally, patients receiving SG experienced longer times to deterioration in global health status (4.3 months vs. 3.0 months) and fatigue (2.2 vs. 1.4 mo). The drug tolerability remained consistent with earlier investigations, albeit with one fatal event [97].

Biomarker analyses indicated that individuals with high/medium Trop-2 levels benefited better from the conjugate independent of their BRCA mutation level, although conclusions about low Trop-2 expression in patients remain uncertain [58]. Subgroup analyses from the ASCENT study underscore the significant advantages of SG in addressing the needs of high-risk populations with mTNBC. Among patients aged ≥65, SG established markedly superior outcomes in comparison with TPC, with PFS of 7.1 vs. 2.4 mo and OS of 14.7 vs. 8.9 mo. Further sub-analysis highlighted its efficacy in Black participants, showing notable improvements in PFS and OS (PFS: 5.4 vs. 2.2 mo, OS: 13.8 vs. 8.5 mo), despite typically poorer outcomes [98,99]. Another sub-analysis of the ASCENT trial specifically evaluated outcomes based on whether patients had TNBC at their initial diagnosis. SG demonstrated notable benefits, with an mPFS of 4.6 vs. 2.3 mo for TPC, and mOS of 12.4 vs. 6.7 mo. The ORR was 31% for SG compared to 4% for TPC. The efficacy and safety profiles appeared comparable between populations with and without TNBC at first diagnosis; emphasizing the need for subtype reanalysis in mTNBC for optimal therapeutic interventions [100].

SG demonstrated modest but promising results in patients with brain metastases, a particularly challenging population. In individuals with stable brain metastases, SG showed a partial response in 3% of individuals and provided comparable safety to the broader study population. In those with undiagnosed brain metastases, SG significantly improved PFS (5.7 vs. 1.5 mo) and OS (10.9 vs. 4.9 mo) compared to standard therapies [101]. In HER2^+^ BC with brain metastases (BCBM) patients, the mOS varies between 30 and 38 mo, contingent upon whether CNS metastases are present at initial diagnosis or arise subsequently [102]; while for HR^+^ BCBM, mOS was 12.5 months [103]. A related trial (NCT03995706) confirmed SG’s CNS activity, demonstrating good tumor penetration and tolerability in Trop-2 expressing brain metastases, with mOS of 35.2 mo in a subset of breast cancer patients undergoing craniotomy [104]. However, the inadequate sample sizing in these studies highlights the want for further research into SG’s role in managing CNS disease.

### 2.5. SG Dosage, Safety, and Management Strategies

#### 2.5.1. Dosage and Administration

SG is infused at 10 mg/kg, the FDA-recommended dose for mTNBC, as it offers increased efficacy with manageable toxicity [89]. SG is prepared as a 180 mg injection, reconstituted in 20 mL sterile saline, diluted to 1.1–3.4 mg/mL, and infused intravenously two times in a 21-day cycle. For patients over 178 kg, doses are split into two infusions, and adjustments are made for weight changes exceeding 10%. The first dosage is administered over a 3 h period, with the patient continuously monitored during the treatment and for 30 min afterward. Subsequently, the drug can be given over a 1–2 h period, with a similar observation period. Infusion rates can be increased gradually if no Grade 1 hypersensitivity reactions occur. The starting infusion rates vary but may begin at 50 mg/h and increase to a peak of up to 1000 mg/h in later doses [82,83,90].

#### 2.5.2. Adverse Events (AEs)

Common AEs in clinical trials included nausea (67%), neutropenia (64%), diarrhea (62%), fatigue (55%), anemia (50%), and vomiting (49%). Grade ≥3 AEs were predominantly neutropenia (42%), with febrile neutropenia occurring in 8% of cases. Phase II studies reported similar safety profiles with rare fatalities (e.g., neutropenic colitis). Hypersensitivity reactions were reported in 37% of individuals, with Grade 3–4 AEs in 2% of populations. Dosage diminutions were done in 34% of cases, and therapy interruptions were common due to neutropenia [56,57,61,83,84]. In a phase II study (NCT04230109) that is still underway, frequently observed AEs included nausea, fatigue, alopecia, neutropenia, and rash.

In a pooled safety examination of 1063 participants given SG 10 mg/kg across four studies (ASCENT, TROPiCS-02, TROPHY-U-01, and IMMU-132-01), neutropenia was reported in 75% of cases, with Grade 3–4 neutropenia occurring in 49%, and febrile neutropenia in 6%, typically with a median onset of 16 days. Neutropenic colitis was reported in 1.4% of individuals. Neutropenia was the leading cause of dose interruptions with 1% discontinued treatment due to neutropenia-related AEs [105].

#### 2.5.3. Dosage Modifications and AE Management

For grade ≥3 AEs, the SG dose is reduced by 25% after the first occurrence and 50% after the second, with treatment discontinuation after three severe events. In cases of Grade 3–4 neutropenia, therapy is paused for 2–3 weeks to allow hematologic recovery before resuming treatment [82]. SG management also includes withholding doses for Grade 3–4 diarrhea, nausea, or vomiting until resolved to ≤Grade 1 (Table 4). For diarrhea, initiate loperamide (4 mg initially, then 2 mg per episode, up to 16 mg daily) after ruling out infections, and discontinue 12 h post-resolution, with supportive care as needed. For hypersensitivity reactions, pre-infusion medication and close monitoring during and 30 min post-infusion are recommended, with emergency treatment readily available. SG is emetogenic, necessitating premedication with a 2- or 3-drug regime to prevent therapy-associated nausea and vomiting [106].

For proactive AE management of neutropenia, the use of granulocyte colony-stimulating factor (G-CSF) is crucial to optimize patient safety and treatment outcomes. If neutropenia happens on day 1 of the treatment cycle, G-CSF (e.g., filgrastim, etc.) once in 24 h for 2–3 consecutive days can be initiated. Alternatively, for neutropenia arising on day 8, pegylated G-CSF (e.g., pegfilgrastim, etc.) may be infused 24–48 h post-therapy to manage the condition effectively [61,107].

Across four SG studies, the usage of G-CSF for prophylaxis or therapy ranged from 30% to 54%. Exploratory analyses indicated that G-CSF prophylaxis reduced the incidence and delayed the onset of Grade ≥3 neutropenia compared to patients without prophylaxis. However, 9% of patients treated with G-CSF for neutropenia still required dose reductions, underscoring the importance of this approach in managing neutropenia-related challenges during SG therapy [54,108,109].

The PRIMED study (NCT05520723) further demonstrated the benefits of prophylactic G-CSF and loperamide in reducing SG-related AEs in advanced TNBC and HR^+^/HER2^−^ BC. Among 50 patients, the reports of ≥Grade 3 neutropenia was 16%, and ≥Grade 2 diarrhea occurred in 16% (including 4% Grade 3), with no febrile neutropenia reported. AE-related diminution and interruption of dosages occurred in 18% and 44% of patients, respectively, but no drug discontinuation was observed. These findings highlight that prophylactic measures improve SG tolerability by reducing severe AEs and minimizing treatment disruptions, thus preserving its therapeutic efficacy [110].

#### 2.5.4. Pharmacogenomics and Personalized Care

Patients with UGT1A1 polymorphisms, particularly homozygous *28/*28 or *6/*6 alleles, are at increased risk of severe neutropenia (Grade 4: 26% vs. 11% in wild-type) [111,112]. Routine UGT1A1 genotyping is recommended to guide dose adjustments and supportive measures, such as growth factor therapy [77,95]. The dose of SG (8 mg/kg vs. 10 mg/kg) does not significantly impact the incidence of AEs across different UGT1A1 genotypes, which suggests that genotype-driven dose adjustments may not be necessary [111,112]. However, while routine genotyping is not yet standard practice, the recommendation for close monitoring in patients with identified UGT1A1 variations is prudent; especially given the clear association between these variations and increased AE risk [113]. Integrating UGT1A1 genotyping into clinical workflows, thus, remains crucial for optimizing SG treatment, balancing efficacy, and minimizing adverse effects. Personalized approaches based on UGT1A1 status could enhance the safety and effectiveness of SG, making it a vital step in advancing patient-centric oncology care.

Exposure–response models from the IMMU-132-01 and ASCENT investigations identified the average SG concentration (CAVG_SG_) as a key predictor of safety outcomes in mTNBC. In the 10 mg/kg dose cohort, model-predicted rates of any-grade AEs were 35.9% for vomiting, 67.4% for diarrhea, 64.7% for nausea, and 67.1% for neutropenia. Higher CAVG_SG_ significantly increased the likelihood of AEs, with neutropenia being the only AE linked to grade ≥3 severity. SG exposure also correlated with increased risks of dose reductions and delays, though the effect of body weight on dose delays was inconclusive, warranting further investigation. These findings highlight the importance of exposure in optimizing SG therapy [114].

### 2.6. Ongoing Trials

Plenty of investigations are evaluating SG across different subtypes of BC either as a stand-alone therapy or in conjunction with other inhibitor molecules. The NeoSTAR trial (NCT04230109) is a phase 2, single-cohort investigation examining the safety and efficacy of SG ± pembrolizumab as neoadjuvant therapy in localized BC across multiple subtypes (Table 5). Cohort A1 focused on 50 treatment-naïve patients with TNBC receiving SG monotherapy. Among these individuals, the pCR rate was 30%, and the ORR was 64%. At a median follow-up of 18.9 mo, the two-year event-free survival (EFS) was 95%, with 100% EFS observed in patients who achieved pCR. Although baseline TROP2 expression could not predict pCR, higher Ki-67 levels and the presence of TILs were prognostic. SG displayed a manageable tolerability in this cohort. Widespread AEs included nausea (86%), fatigue (84%), alopecia (76%), and neutropenia (58%). Grade ≥3 AEs primarily involved neutropenia, diarrhea, leukopenia, fatigue, and nausea. Only 6% of patients required dosage reductions and no stoppage of therapy due to AEs or disease progression were reported [115,116].

The SASCIA study (NCT04595565) is a phase 3, international, multicenter study comparing SG to NACs in individuals with HER2^−^ BC who have residual disease post-NAC. The main recorded outcome is invasive disease-free survival (iDFS), with OS and subgroup analyses as secondary endpoints. Treatment continuation rates were 75% for the SG cohort and 65.6% for the TPC arm. AEs were frequent with SG, including six serious adverse events (SAEs), such as blood and lymphatic system disorders and infections. The rates of dosage modifications were consistent across treatment groups, and no unforeseen AEs or toxicities were identified with SG. Consequently, the independent data monitoring committee endorsed proceeding with the study without any adjustments [117,118].

The active, not recruiting EVER-132-001 study (NCT04454437), is evaluating the efficiency and safety of SG in 80 Chinese individuals with relapsing mTNBC and having undergone two previous drug regimens. The study found an ORR of 38.8% and a CBR of 43.8%, with a mPFS of 5.6 mo and mOS of 14.7 mo. Altogether, SG exhibited significant efficacy and relatively controllable tolerability in this extensively pretreated group [119].

SWOG S2007 (NCT04647916) is an ongoing phase II investigation evaluating the intracranial (IC)-ORR of SG for HER2^−^ BCBM individuals, including those with active metastases [120]. Other studies are examining SG in combination with pembrolizumab (ASCENT-04, NCT05382286 [121], and ASCENT-05, NCT05633654 [122]) for eight cycles versus pembrolizumab and TPC. The ASCENT studies focus on patients with all types of PD-L1-positive TNBC.

The Morpheus-panBC investigation examines various drug combinations for first-line therapy of untreated localized advanced breast cancer (ABC) or metastatic breast cancer (mBC). Preliminary findings of the atezolizumab and SG groups showed an ORR of 76.7% compared to 66.7% with atezolizumab plus nab-paclitaxel. The atezo + SG arm showed a higher CBR (83.3% vs. 66.7%), with 5 complete responses. PFS data, though immature, suggested a benefit with atezolizumab and SG (12.2 vs. 5.9 mo) [123,124]. Despite the promising activity, a key limitation is the omission of individuals with active brain metastasis, highlighting the need for further research on CNS efficacy in immunotherapy combinations.

Ongoing research is exploring additional combination therapies involving SG. These include combining SG with hydroxychloroquine (HCQ) for BC (NCT06328387) and pairing SG with loperamide and G-CSF to improve its tolerability in patients with mTNBC (NCT05520723). These approaches aim to enhance clinical efficacy and offer more efficient targeted therapeutic options. Further, a TROPiCS-04 study (NCT04527991) is an ongoing open-label, global, phase III investigation studying the efficacy and safety of SG versus TPC in pre-treated patients. No changes to SG’s known safety profile have been observed for its approved BC indications or investigational uses. However, integrating more references or data points from ongoing studies would solidify the future outlook of SG and demonstrate how SG is shaping the evolving ADC landscape.

### 2.7. Comparison of SG with Other ADCs and Its Sequencing Strategies

Mechanistically, SG delivers SN-38 to Trop-2^+^ tumors with a unique bystander effect, enhancing efficacy in heterogeneous cancer environments compared to other ADCs; thus strengthening its unique positioning in oncology. However, the narrative could benefit from more balanced comparisons with other ADCs. For example, including specific data on competing therapies would provide a clearer picture of SG’s relative advantages and limitations. However, the approvals of ADCs such as trastuzumab deruxtecan (T-Dxd) based on studies involving varying HER2 expression levels and prior lines of chemotherapy [125], complicate efficacy comparisons. In addition, while SG’s role in treatment algorithms is well-highlighted, further exploration of its positioning in combination regimens or potential use in earlier lines of therapy could enhance the discussion.

Datopotamab deruxtecan (Dato-DXd), another ADC targeting TROP2 with a TOPO-I inhibitor payload, was evaluated in the phase I TROPION-PanTumor01 investigation for HR^+^/HER2^–^ BC and TNBC, demonstrating ORRs of 26.8% and 31.8%, mPFS of 8.3 and 4.4 mo, and an mDOR of 16.8 mo for TNBC. Stomatitis was the most common adverse event, observed in 50% of patients [126]. The TROPION-Breast01 study faced limitations, including shifts in HR^+^ mBC treatment, patient-driven imbalances in the open-label design, and inconsistent use of prophylactic mouthwash, complicating stomatitis assessment. Further research is needed to address these challenges and optimize ADC use.

Variations in ADC design, including target antigens, linkers, payloads, and drug-to-antibody ratios, contribute to differences in safety outcomes [127,128]. For instance, SG’s lower serum stability leads to higher systemic toxicity [89,129] compared to Dato-DXd, which has a stable linker releasing minimal payload in plasma [130]. Hematologic toxicities, like neutropenia, were frequent with SG (51% grade ≥3) in TROPiCS-02 but less common with Dato-DXd in TROPION-Breast01 [130]. In an early-phase investigation, sacituzumab tirumotecan (SKB264/MK-2870, ST) frequently caused grade ≥3 hematological reactions, including decreased neutrophil count (37%), lymphocyte count (22%), and anemia (15%) [131]. Diarrhea (9% grade ≥3) and stomatitis rates also varied between TROP2-directed ADCs, with Dato-DXd showing milder profiles compared to ST and SG [130,131].

Both SG and T-DXd improve mOS by 5–7 mo compared to chemotherapy [58,61] and exhibit bystander effects in heterogeneous tumor environments [132,133]. T-DXd’s deruxtecan payload is a far superior TOPO-I inhibitor than SG’s SN-38, and its bystander outcome is critical in low HER2 environments, whereas SG’s reliance on this effect is less clear due to high Trop-2 expression [134]. Despite differing targets and structures, both ADCs share TOPO-I inhibitor payloads, raising questions about sequential efficacy.

A case report demonstrated the clinical outcome of T-DXd after SG therapy. Three years post-NAC, a female with recurrent BC and HER2 IHC 1+ experienced a 14-month recovery to SG, followed by an 11-month response to T-DXd with manageable side effects, before proceeding to capecitabine [135]. This study emphasizes the need for repeated HER2 testing due to expression heterogeneity and treatment implications. Additional research is required to measure responses, investigate resistance, and evaluate ADC sequencing strategies.

An ADC-Low multicenter retrospective study compared 179 patients with HR^+^/HER2^low^ or HR^−^/HER2^low^ mBC treated with SG and T-DXd in either sequence, with or without intermediate NAC. Among them, 64.2% received SG first, and 58.1% transitioned directly to the second ADC (ADC2). With a median follow-up of 6 mo, 68.2% discontinued ADC2, primarily due to disease progression. The mPFS with ADC2 was 2.7 mo (2.2 mo for HR^+^/HER2^low^ individuals receiving SG following T-DXd therapy, and 3.1 mo for HR^−^/HER2^low^ patients receiving T-DXd after SG), with minimal variation based on the treatment order [136]. However, a subset of patients demonstrated initial short-term responses, indicating that while the overall clinical benefit of this sequential approach is limited, further investigations are warranted to categorize subgroups that might profit from this approach.

Two US-based studies examined the outcomes of sequential ADC use in BC. The first multi-institution study analyzed individuals with HR^+^/HER2^−^ mBC or TNBC who received at least two ADCs. The mPFS was 5.3 mo with ADC1 and 2.5 mo with ADC2. In TNBC, PFS was 2.7 mo for T-DXd after SG, while HR^+^/HER2^−^ individuals receiving SG post T-DXd had a PFS of 1.6 mo. Cross-resistance to the second ADC occurred in 59.4% of cases, with higher rates observed when the second ADC targeted the same tumor-associated antigen (78.5%) compared to a different one (53.1%), regardless of payload similarity. Tumor sequencing revealed *TOPO-I* gene mutations linked to resistance, underscoring the need for biomarker-driven ADC sequencing strategies in mBC [137,138]. These findings highlight the heterogeneity of resistance mechanisms and suggest biomarker-driven strategies may optimize ADC sequencing in mBC.

Another study in the USA analyzed the sequential treatment of HER2^low^ mBC individuals with T-DXd and SG. HR^+^/HER2^low^ individuals showed better outcomes with T-DXd after SG (mPFS: 3.7 mo, ORR: 34.8%) compared to SG after T-DXd (PFS: 2.6 mo, ORR: 18.5%). For HR^-^/HER2^low^ MBC, T-DXd after SG yielded a PFS of 2.8 mo and ORR of 35.0%. Safety evaluations highlighted frequent dose reductions (46.4% for SG, 15.5% for T-DXd) and treatment discontinuations due to toxicity (8.3% SG, 10.7% T-DXd). Significant toxicities included T-DXd-associated interstitial lung disease (ILD)/pneumonitis (16.7%, grade ≥3 in 4.8%) and SG-related neutropenia delays (17.9%) [139]. An ongoing SATEEN study (NCT06100874) seeks to assess the risk and benefit of SG when combined with trastuzumab for HER2^+^ mBC individuals. The investigation will provide insights into the medical benefits of SG in non-HER2 receptor-negative BC. The results underline the challenges and limited benefits of sequential ADC use in heavily pretreated BC patients.

A recent retrospective investigation highlighted the real-world outcome of SG and T-DXd in treating BCBMs, including both stable and active cases. Among 16 patients, SG demonstrated an intracranial disease control rate (icDCR) of 42% and a median intracranial progression-free survival (icPFS) of 2.7 mo, with mOS of 6.4 mo. T-DXd achieved an icDCR of 88%, with a median icPFS of 11.2 mo and OS of 27.1 mo. The activity of T-DXd was observed even in individuals treated after SG, suggesting the feasibility of sequential ADC use. While HER2^+^ patients had better outcomes than HER2^low^ patients, active and stable BCBMs showed no significant differences in outcomes for either ADC [140]. Another recently published analysis of mBC patients receiving at least two ADC types found that SG-containing regimens showed numerically longer PFS (9.10 vs. 6.35 mo) and OS (31.9 vs. 22.0 mo) compared to HER2 ADCs in HER2^low^ disease, though differences were not statistically significant. In patients who progressed on SG, outcomes with subsequent T-DXd were similar [141]. Although the safety profiles of both studies were consistent with prior data, small sample sizes, heterogeneous patient populations, non-standardized imaging criteria, inadequate genomic data, and reliance on prior histology for tumor biology in most cases, limit their broad applicability. The retrospective design also introduces potential biases, reducing comparability with prospective trials and restraining the ability to establish causality.

### 2.8. Cost Evaluation of Sacituzumab Govitecan

A 50-mL, 180 mg injection of SG is available in the market for $1478.00/injection, while the estimated cost per 21-day treatment cycle is $12,478, based on the assumption of 94% dosage strength and a mean body weight of 71.1 kg [142]. Univariate analyses showed that outcomes are particularly responsive to the pharmacological cost of SG and the utilities of PFS and advanced malignancy. Base-case calculations reveal that the incremental cost-effectiveness ratio (ICER) of SG in contrast to conventional drug therapy surpasses the US willingness-to-pay (WTP) benchmark of $150,000/QALY, rendering it cost-ineffective. In the overall mTNBC population, SG costs an additional $293,037 and provides 0.2340 more QALYs, resulting in an ICER of $1,252,295 per QALY (quality-adjusted life year) [143]. Based on the Canadian Agency for Drugs and Technologies in Health (CADTH) reevaluation, the ICER for SG vs. TPC is $375,333 per QALY gained. SG must reduce its price by at least 87% to be affordable as per the WTP criterion of $50,000 per QALY threshold [142].

### 2.9. Drug Resistance to Sacituzumab Govitecan

Due to the complexity and variability of target antigens and payloads, ADC resistance mechanisms are diverse and may require individualized study for each ADC. Emerging issues following the approval of SG for HR^+^/HER2^−^ and mTNBC include the need for a deeper understanding of newly developed resistance to the SG conjugate and strategies to overcome it [144]. Further, understanding the mechanisms of resistance is also critical in the consideration of the therapy sequence.

A key factor in resistance to ADCs arises from either downregulation or mutation of the receptor targets, reducing payload delivery [145]. Genomic analysis of a TNBC patient with under-expressed Trop-2 revealed mutations in *TOPO-I* (topoisomerase-1) and *TACSTD2* (Trop-2), correlating with developing resistance against SG [146]. Individuals with reduced Trop-2 expression had shorter median PFS when treated with SG versus those with elevated expression (2.7 months vs. 6.9 months) [58]. Additionally, research showed that tamoxifen treatment drastically improved Trop-2 levels [147]. One strategy to overcome ADC resistance is upregulating target antigen expression. Until now, temporal variations in Trop-2 expression during malignancy and therapy are not fully clear, indicating a potential arena for future research.

A recent study highlights the potential for cross-resistance between ADCs in mBC, especially when the same antibody target is used in consecutive treatments. While switching targets showed some improvement in PFS, the results were not statistically significant, suggesting the need for further investigation. Mechanisms like altered antigen expression or drug resistance may explain the reduced efficacy of subsequent ADCs. This study emphasizes the importance of optimizing ADC sequencing and exploring biomarker-driven approaches to improve patient outcomes and overcome resistance [148]. As such, success in addressing resistance will depend on a deeper understanding and evaluation of these mechanisms in diseased population.

## 3. Future Directions

SG is constantly being evaluated in clinical investigations, both as a monotherapy and in conjunction with PARPi, chemotherapeutic agents, immunotherapies, across multiple cancer types, indicating that its usage may extend beyond third-line treatment for mTNBC and HR^+^ BC. The outcomes of these trials will offer significant insights into SG’s potential across various clinical settings.

Combinations of SG with other cytotoxic drugs show promising antitumor potential. For example, using ATP binding cassette subfamily G member 2 (ABCG2) inhibitors with SG has been effective in overcoming resistance to SN-38 in various neoplasms [149]. The rationale for combining SG with immunotherapy is strong, as ADCs can induce immunogenic cell death and T cell infiltration, while ICIs restore exhausted T lymphocytes, which result in synergistic outcomes.

Preclinical studies have already supported the synergy between SG and PARPi in TNBC, and further clinical trials are anticipated [150]. The ORR was 30.1%, the 6-month CBR 53.8%, and mPFS and OS were 6.2 and 18.0 mo, respectively. Common adverse events included neutropenia (88.5%) and anemia (92.3%), with dose reductions in 58% of patients. The combination demonstrated feasibility and efficacy, supporting further randomized trials [151]. Further, the BEGONIA Arm 6 evaluated the efficacy of durvalumab combined with T-DXd in participants with ER^−^/PR^−^/HER2^low^ advanced breast cancer (ABC). After a median follow-up of 10.1 mo, the ORR was 57%, with responses observed in both PD-L1^high^ and PD-L1^low^ subgroups. This combinatorial approach presented a tolerable safety score [152]. Still, the cumulative toxicity of combinatorial approaches remains a significant concern. Thus, research on Trop-2-targeted ADCs should focus on better patient selection, optimizing ADC design for improved safety and efficacy, and overcoming resistance to maximize treatment benefit [153].

Further, new strategies beyond traditional ADCs, such as immune-conjugates and nano-structures, are still in infancy. Liu et al. created a Trop-2-directed tetrakis-ranpirnase conjugate (IgG-Rap immunoRNase) using the hRS7 mAb, which demonstrated significant survival benefits in TNBC mouse models [154]. Next-generation anti-Trop-2 therapies are being developed to leverage different ADC linker structures, conjugation chemistries, and more potent drug payloads [155]. A novel ADC strategy involves the use of bispecific antibodies targeting distinct epitopes on each F_ab_ combined with an alternative payload to overcome resistance to cytotoxic therapies. This bispecific ADC (zanidatamab zovodotin) targeting HER2 with trastuzumab/pertuzumab F_ab_ specificities and an auristatin payload, showed a 28% ORR in a phase 1 trial for HER2^+^ABC [156].

Follow-up research will likely concentrate on identifying and validating predictive biomarkers, with a particular emphasis on investigating the interplay between Trop-2 expression levels, UGT1A128 homozygosity, and clinical outcomes in cancer treatment [111]. Understanding how variations in Trop-2 expression correlate with UGT1A128 genetic polymorphisms may provide insights into individual patient responses to therapies targeting Trop-2, such as SG. Ultimately, such investigation could elucidate mechanisms of treatment efficacy and toxicity, enabling the development of more personalized treatment strategies that enhance therapeutic effectiveness while minimizing AEs and contribute to improved clinical outcomes in cancer management.

Future cancer therapies targeting the Trop-2 protein will likely prioritize strategies that selectively target the “cleaved” or activated form of Trop-2 present in neoplasms [157,158], while sparing the intact form expressed in normal tissues. This approach has the potential to enhance treatment efficiency whilst reducing off-target binding and diminishing toxicity, thus increasing the therapeutic window of Trop-2-targeted interventions.

## 4. Conclusions

The successful application of SG in mBC demonstrates that Trop-2-directed therapeutics is a promising avenue for exploration. However, challenges associated with existing targeted therapies illustrate the need for continued refinement and optimization of treatment strategies. Advances in the structural and functional analysis of Trop-2 and its expression patterns could guide the development of next-generation ADCs, enhancing treatment efficacy. Moreover, integrating SG with ICIs or PARPi aligns with the principles of precision medicine by combining therapies that target different aspects of tumor biology, thus aiming to improve clinical outcomes through a multifaceted approach. This strategic combination not only holds promise for improving efficacy but also emphasizes the necessity of personalized treatment regimens that consider individual patient characteristics and tumor profiles.

As ADCs are increasingly integrated into early BC treatment, sequencing and resistance pose significant clinical challenges. Recent data suggest limited efficacy when sequencing ADCs with similar TOPO-I inhibitor payloads, emphasizing cautious use until alternative payloads are available. Without randomized trials comparing ADC sequencing to standard chemotherapy, the impact on survival or quality of life remains unclear.

In conclusion, SG’s comparative advantages lie in its ability to deliver consistent efficacy, manageable toxicity, and applicability across diverse breast cancer subtypes. Its role in sequencing strategies is also being explored to optimize its use alongside or after other ADCs.

## Figures and Tables

**Table 1:** Key characteristics of sacituzumab govitecan (Trodelvy™).

Parameters	Key Features
Brand name	Trodelvy
Generic name	Sacituzumab govitecan-hziy
Company	Gilead Sciences Inc.
Dosage form	Injection
Color	Off-white powder
Mode of action	Antibody targets the Trop2 receptor on the cancer cell membrane SN-38 is released intracellularly as well as extracellularly SN-38 binds to and stabilizes topoisomerase IB
Indication	Advanced TNBC, after at least two lines of systemic treatment (including taxane), one in the metastatic setting Advanced HR^+^/HER2^–^ breast cancer resistant to endocrine therapy, with at least two prior lines of treatments in the metastatic setting
FDA Approval date	22 April 2020 for metastatic TNBC with at least two prior therapies and 3 February 2023 for HR^+^/HER2^–^ breast cancer
Dosage	10 mg/kg intravenously
Maximum tolerated dose	10 mg/kg/day, if weight changes by more than 10%, recalculate the dose
Administration	Premedicate with antipyretics and histamine antagonists Antiemetics for moderately emetogenic drugs on days 1, 8 every 21 days Continue till progressive disease or dose-limiting toxicity
Toxicities %	Neutropenia (boxed warning): 61% Diarrhea (boxed warning): 65% Hypersensitivity: 37%
Molecular predictors of response	No molecular predictors have been validated Testing for Trop2 expression is not needed
Special considerations	To consider UGT1A1 polymorphisms in patients with unusually severe or rapid onset adverse effects
Cost	$12,478 for a single 50-mL, 180 mg vial

**Table 2:** Pharmacokinetic parameters for SG and SN38.

Parameter	SG	SN-38
C_max_, mean (CV%), (ng/mL)	239,000 (11%)	98 (45%)
AUC_0–168_, mean (CV%), (ng·h/mL)	5,640,000 (22%)	3696 (56%)
V_d_ (L)	3.6	-
t_½_ (hours)	16	18
C_L_^a^, L/h	149	270,000
V_ss_^a^, L	2820	6900
Systemic clearance time (h)	11–14	10–20
Mean clearance rate, L/h	0.13	11.2
Median elimination time (h)	23.4	17.6

C_max_, maximum serum concentration; AUC_0–168_, area under the serum concentration curve from time zero to 168 h; V_d_, volume of distribution; t_½_, half-life; C_L_, total body clearance, V_ss_, apparent volume of distribution at steady state following IV administration.

**Table 3:** Landmark clinical trials of sacituzumab govitecan that led to FDA approval.

Clinical Trial	Design	Intervention	Patient Population	No. of Pts.	Response Rates	Median PFS (Months)	Median OS (Months)	Grade ≥ 3 AEs	FDA Approval
ASCENT (NCT02574455) [85]	Phase III RCT	SG vs. single agent CT	Metastatic TNBC refractory to two prior lines (including taxanes)	529	31.1 % vs. 4.2 %	4.8 vs. 1.7	11.8 vs. 6.9	Neutropenia (51%) Leukopenia (10%) Diarrhea (10%) Anemia (8%)	Regulatory approval (7 April 2021)
TROPiCS-02 (NCT03901339) [97]	Phase III RCT	SG vs. single-agent CT	HR^+^/HER2^−^ mBC prior taxane, CDK4/6 inhibitor and 2–4 prior CTs	543	57 % vs. 38 %	5.5 vs. 4.0	13.9 vs. 12.3	Neutropenia (51%) Diarrhea (10%)	Regulatory approval (3 February 2023)

RCT, randomized controlled trial; CT, chemotherapy; CDK 4/6, cyclin-dependent kinase 4/6; OS, overall survival; PFS, progression-free survival; TNBC, triple-negative breast cancer; FDA, Food and Drug Administration; AE, adverse events.

**Table 4:** Dose modifications for adverse reactions.

Adverse Reaction	Occurrence	Dose Modification
**Severe Neutropenia**		
Grade 4 neutropenia ≥7 days OR Grade 3 febrile neutropenia OR Grade 3-4 neutropenia which requires a 2-to-3-week dose delay for recovery to ≤Grade 1	First	Administer G-CSF
	Second	25% dose reduction
	Third	50% dose reduction
	Fourth	Discontinue treatment
Grade 3-4 neutropenia which requires a dose delay longer than 3 weeks for recovery to ≤Grade 1	First	Discontinue treatment
**Severe toxicities other than neutropenia**		
Grade 4 non-hematological toxicity of any duration OR Any Grade 3 nausea OR Any Grade 3-4 vomiting or diarrhea due to treatment that is not controlled with anti-emetics and anti-diarrheal agents OR Other Grade 3 non-aematological toxicity persisting >48 hours despite optimal medical management OR Any Grade 3-4 toxicity (other than neutropenia), which requires a 2- or 3-week dose delay for recovery to ≤Grade1	First	25% dose reduction
	Second	50% dose reduction
	Third	Discontinue treatment
Any Grade 3-4 toxicity (other than neutropenia), which does not recover to ≤Grade 1 within 3 weeks	First	Discontinue treatment

**Table 5:** Ongoing clinical trials of sacituzumab govitecan.

Clinical Trial	Sponsor	Intervention	Phase	N	Status	Population	Primary Endpoints
NCT04230109 (NeoSTAR)	Massachusetts General Hospital	SG with or without pembrolizumab	II	260	Recruiting	Localized TNBC	pCR
NCT04595565 (SASCIA)	German Breast Group	SG vs. TPC	III	1332	Active, not recruiting	Residual disease in HER2^−^ BC post NAC	IDFS
NCT05382299 (ASCENT-03)	Gilead Sciences	SG vs. TPC	III	540	Recruiting	1st line mTNBC	PFS
NCT04647916 (SWOG)	SWOG Cancer Research Network	SG	II	44	Recruiting	HER2^−^ BCBM	IC-ORR
NCT05382286 (ASCENT-04)	Gilead Sciences	SG + pembrolizumab vs. TPC	III	443	Active, not recruiting	First-line mTNBC whose tumors express PD-L1	PFS
NCT05633654 (ASCENT-05)	Gilead Sciences	SG + pembrolizumab vs. capecitabine	III	1514	Recruiting	Residual TNBC disease after surgery and NAC	IDFS
NCT04448886 (Saci-IO HR^+^)	Dana-Farber Cancer Institute	SG with or without pembrolizumab	II	110	Active, not recruiting	1st or 2nd line PD-L1^+^ HR^+^/HER2^−^ mBC	PFS
NCT04468061 (Saci-IO TNBC)	Dana-Farber Cancer Institute	SG with or without pembrolizumab	II	110	Recruiting	First-line PD-L1^−^ mTNBC	PFS
NCT03971409 (InCITe)	University of California	SG + immunotherapy	II	150	Recruiting	mTNBC	ORR
NCT05143229 (ASSET)	University of Kansas Medical Center	SG + aleplisib	I	18	Recruiting	2+L HER2^+^ mBC	RP2D
NCT04434040 (ASPRIA)	Dana-Farber Cancer Institute	SG + atezolizumab	II	40	Active, not recruiting	Residual invasive disease in TNBC following NAC	Undetectable ctDNA
NCT03424005 (Morpheus-panBC)	Hoffmann-La Roche	SG + Atezolizumab	I/II	580	Recruiting	Metastatic or Locally Advanced BC	ORR
NCT04039230	Massachusetts General Hospital	SG + talazoparib	Ib/II	75	Recruiting	mTNBC	DLT, PFS, ORR
NCT06328387 (SYSKY-2024-064-03)	Sun Yat-Sen Memorial Hospital of Sun Yat-Sen University	SG vs. HCQ + SG	I/II	129	Recruiting	ABC	DLT, AEs, ORR
NCT06100874 (SATEEN)	Dana-Farber Cancer Institute	SG vs. SG + Trastuzumab	II	40	Recruiting	HER2^+^ mBC	ORR
NCT05520723 (PRIMED)	MedSIR	SG + Loperamide + G-CSF	II	50	Active, not recruiting	mTNBC	Grade ≥ 2 diarrhea or grade ≥ 3 neutropenia

SG: sacituzumab govitecan; TNBC: triple-negative breast cancer; pCR: pathologic complete response; TPC: treatment of physician’s choice; BC: breast cancer; IDFS: invasive disease-free survival; mTNBC: metastatic TNBC; PFS: progression-free survival; BCBM, breast cancer with brain metastasis; IC-ORR, intracranial objective response rate; PD-L1: programmed death ligand-1; NAC: neoadjuvant chemotherapy; mBC: metastatic breast cancer; ORR: overall response rate; 2+L: two or more lines of therapies; RP2D, recommended phase II dose; ctDNA: circulating tumor DNA; DLTs: dose-limiting toxicities; HCQ, hydroxychloroquine; ABC, advanced breast cancer; AE, adverse event; G-CSF, granulocyte colony-stimulating factor.

## Data Availability

All data are available within the manuscript.
